# Reduced physical activity in young and older adults: metabolic and musculoskeletal implications

**DOI:** 10.1177/2042018819888824

**Published:** 2019-11-19

**Authors:** Kelly A. Bowden Davies, Samuel Pickles, Victoria S. Sprung, Graham J. Kemp, Uazman Alam, Daniel R. Moore, Abd A. Tahrani, Daniel J. Cuthbertson

**Affiliations:** School of Biomedical, Nutritional and Sport Sciences, Faculty of Medical Sciences, Newcastle University, Catherine Cookson Building M1.038, Newcastle upon Tyne, NE2 4HH, UK; Institute of Ageing and Chronic Disease, University of Liverpool, Liverpool, UK; Institute of Ageing and Chronic Disease, University of Liverpool, Liverpool, UK; Obesity and Endocrinology Research Group, Aintree University Hospital NHS Foundation Trust, Liverpool, UK; Research Institute for Sport and Exercise Science, Liverpool John Moores University, Liverpool, UK; Institute of Ageing and Chronic Disease, University of Liverpool, Liverpool, UK; Obesity and Endocrinology Research Group, Aintree University Hospital NHS Foundation Trust, Liverpool, UK; Institute of Ageing and Chronic Disease, University of Liverpool, Liverpool, UK; Liverpool Magnetic Resonance Imaging Centre (LiMRIC), University of Liverpool, Liverpool, UK; Institute of Ageing and Chronic Disease, University of Liverpool, Liverpool, UK; Obesity and Endocrinology Research Group, Aintree University Hospital NHS Foundation Trust, Liverpool, UK; Pain Research Institute, University of Liverpool, Liverpool, UK; Division of Endocrinology, Diabetes and Gastroenterology, University of Manchester, Manchester, UK; Department of Diabetes and Endocrinology, Royal Liverpool and Broadgreen University NHS Hospitals Trust, Liverpool, UK; Faculty of Kinesiology and Physical Education, University of Toronto, Toronto, ON, Canada; Institute of Metabolism and Systems Research, College of Medical and Dental Sciences, University of Birmingham, Birmingham, UK; Centre of Endocrinology, Diabetes and Metabolism (CEDAM), Birmingham Health Partners, Birmingham UK; Department of Diabetes and Endocrinology, University Hospitals Birmingham NHS Foundation Trust, Birmingham, UK; Institute of Ageing and Chronic Disease, University of Liverpool, Liverpool, UK; Obesity and Endocrinology Research Group, Aintree University Hospital NHS Foundation Trust, Liverpool, UK

**Keywords:** anabolic resistance, body composition, insulin resistance, liver fat, physical inactivity, skeletal muscle

## Abstract

**Background:**

Although the health benefits of regular physical activity and exercise are well established and have been incorporated into national public health recommendations, there is a relative lack of understanding pertaining to the harmful effects of *physical inactivity*. Experimental paradigms including complete immobilization and bed rest are not physiologically representative of sedentary living. A useful ‘real-world’ approach to contextualize the physiology of societal downward shifts in physical activity patterns is that of short-term daily *step reduction*.

**Results:**

Step-reduction studies have largely focused on musculoskeletal and metabolic health parameters, providing relevant disease models for metabolic syndrome, type 2 diabetes (T2D), nonalcoholic fatty liver disease (NAFLD), sarcopenia and osteopenia/osteoporosis. In untrained individuals, even a short-term reduction in physical activity has a significant impact on skeletal muscle protein and carbohydrate metabolism, causing anabolic resistance and peripheral insulin resistance, respectively. From a metabolic perspective, short-term inactivity-induced peripheral insulin resistance in skeletal muscle and adipose tissue, with consequent liver triglyceride accumulation, leads to hepatic insulin resistance and a characteristic dyslipidaemia. Concomitantly, various inactivity-related factors contribute to a decline in *function*; a reduction in cardiorespiratory fitness, muscle mass and muscle strength.

**Conclusions:**

Physical inactivity maybe particularly deleterious in certain patient populations, such as those at high risk of T2D or in the elderly, considering concomitant sarcopenia or osteoporosis. The effects of short-term physical inactivity (with step reduction) are reversible on resumption of habitual physical activity in younger people, but less so in older adults. Nutritional interventions and resistance training offer potential strategies to prevent these deleterious metabolic and musculoskeletal effects.

**Impact:**

Individuals at high risk of/with cardiometabolic disease and older adults may be more prone to these acute periods of inactivity due to acute illness or hospitalization. Understanding the risks is paramount to implementing countermeasures.

## Introduction

The spectrum of bodily movement spans sleep, bed rest and sitting through to light, moderate and vigorous physical activity (PA), all of which stress differing physiological pathways. Exercise physiology research has irrefutably demonstrated the benefits of regular exercise. However, more recently researchers have turned their attention to the other end of the spectrum, examining the harmful effects of physical inactivity and sedentary behaviour, which more accurately represent Western societal lifestyle norms. Based on recent consensus,^1^ these key terms are defined as ‘performing insufficient amounts of moderate- and vigorous- intensity PA (MVPA), i.e. not meeting specified physical activity guidelines’ and ‘any waking behaviour characterised by an energy expenditure ⩽1.5 METs’, respectively. It has become clear that the whole body, tissue-specific and cellular responses to physical inactivity and sedentary behaviour are not simply opposites of those to exercise. In fact, it has been suggested that the gene–lifestyle mismatch of today’s sedentariness compared with our evolutionary ancestors would mean exercise, or those who are ‘trained’, represents the normal biologically healthful state, whereas the lack of exercise or inactivity ultimately precipitates a diseased state.^2^

Much indirect evidence examining the detrimental effects of physical inactivity has come from epidemiological studies, although there is increasing interest in experimental studies of reduced human movement. Although bed rest,^3^ limb immobilization^4^ and cessation of exercise in trained volunteers^5^ can provide valuable information on the deleterious effects of inactivity, this review will not cover these extreme experimental models as their translation to the more ‘benign’ forms of inactivity in free-living environments is difficult. This review will instead focus upon a more physiologically representative model of reduced PA, that of short-term step reduction, whereby daily activity patterns are modified to reduce PA levels and increase sedentary behaviour. We believe this model more adequately mimics acute illness and hospitalization as well as the societal changes in PA patterns that have occurred with related changes in technology, culture and work patterns.^6^ By scrutinizing this arguably more ecologically valid research model, we can gain improved mechanistic insight into the metabolic and musculoskeletal dysfunction that occurs with real-life physical inactivity. Furthermore, it is important to consider our ageing society. Evidence has shown the harmful effects of lifelong sedentarism but acute periods of physical inactivity must also be considered as these are arguably more detrimental to the health of older adults when compared to young people.

## Epidemiological evidence on the benefits of physical activity

Since the seminal observations of Morris and colleagues in the 1950s, who demonstrated a higher risk of coronary heart disease in bus drivers (physically inactive, seated during their working day) *versus* bus conductors (physically active, walking up and down aisles and stairs), the health benefits of PA have become increasingly clear.^7^ Regular exercise and/or PA has been demonstrated to instigate beneficial effects in obesity, metabolic syndrome, type 2 diabetes (T2D), nonalcoholic fatty liver disease (NAFLD), cardiovascular disease (CVD), some cancers, and to reduce overall mortality, which is often independent of weight loss.^8,9^ It is also important in musculoskeletal health, maintaining bone and skeletal muscle mass as well as attenuating the major features of ageing.^10,11^ Thus, it is unsurprising that physical fitness, which ultimately is related to total PA levels, is generally regarded as the single best predictor of all-cause mortality, independent of disease state.^12^

Conversely, the relationship between *physical inactivity* and major noncommunicable diseases has been well-evidenced globally.^13,14^ Public health activity guidelines emphasize the importance of PA, recommending 150 min of moderate to vigorous physical activity (MVPA) per week, 5 days per week. However, one-quarter of the UK population are failing to achieve even 30 min of moderate activity per week and 90% of the American population do not achieve PA recommendations.^15^ In 2006/2007, the estimated cost of physical inactivity in the UK to the NHS was £900 million.^16^ Compared with the substantial body of work investigating the acute and chronic effects of exercise, relatively little is understood regarding the mechanistic changes that result from physical inactivity. PA in Western society has changed drastically since the Industrial Revolution, with ample evidence for a reduction in occupational energy expenditure as well as greater time spent sitting.^17,18^ Therefore, additional efforts need to be directed to better understand the deleterious effects of physical inactivity.

## A brief overview of the harmful effects of low physical activity and sedentary behaviour

Physical inactivity and sedentary behaviour contribute to low levels of energy expenditure and are associated with many detrimental effects, including loss of aerobic fitness and musculoskeletal and cognitive decline. Sedentary time can account for 60% of waking hours (6–10 h/day) and behaviours such as TV viewing are associated with increased risk of all-cause and cardiovascular mortality independent of smoking, hypertension, hypercholesterolemia and diet.^19,20^ Studies examining the physiological impact of ‘lack of human movement’ in contrast to physical activity/exercise, the two opposite ends of the activity spectrum (i.e. sedentary behaviour to MVPA), are not necessarily mutually exclusive. It is now clear that increased sedentary time is distinct from too little activity, and is itself associated with an independent increased risk of all-cause and cardiovascular mortality.^19^ While the benefits of meeting the PA guidelines are not disputed, minimizing sedentary time is of vital importance. To elucidate the relationship between the apparently distinct categories of PA and sedentary time, Yates and colleagues studied ~2000 adults using accelerometry, categorizing them into four groups: ‘Busy Bees’ – physically active and low sedentary time; ‘Sedentary Exercisers’ – physically active and high sedentary time; ‘Light Movers’ – physically inactive and low sedentary time; and ‘Couch Potatoes’ – physically inactive and high sedentary time.^21^ Being physically active was associated with a better anthropometric and metabolic health profile whereas being sedentary independent of activity (Light Movers) is associated with lower HDL (high-density lipoprotein) cholesterol (a traditional cardiovascular risk factor). Sedentary time is associated with negative health outcomes, independent of PA^22^ and MVPA.^23^ Furthermore, levels of physical activity/fitness are correlated with liver and visceral fat accumulation in individuals at high risk of T2D^24^ and normal-weight healthy adults.^25^

## Metabolic effects of physical inactivity/step reduction

Bed rest and disuse/unloading models induce deleterious metabolic effects, including increased insulin resistance and inflammation, as well as alterations to insulin signalling, adipose tissue lipolysis and mitochondrial pathways.^3–5^ Likewise, step reduction can lead to a number of metabolic maladaptations in healthy adults (Table 1), though direct comparisons between studies can be difficult due to differences in the baseline PA level (ranging from ‘highly active’ being >6000 to >10,000 steps/day), the level of ‘low’ step count (ranging from <1500 to <5000), the duration of reduced activity (ranging from 3 to 14 days) and the additional effect of overfeeding.^26,27^ When focusing on studies in which individuals transition from well-defined high levels of PA (~10,000 steps/day) to low (~1500 steps/day) for ⩾14 days, the results are striking, especially when one considers the volunteers were young, healthy adults.^28–30^ Two studies have administered step reduction with overfeeding,^26,27^ with an amplified effect.

**Table 1.** A summary of intervention studies examining the effects of reduced physical activity and increased sedentary behaviour in younger adults (18–65 years old). The table outlines the participant cohorts (including age and BMI), details of the inactivity intervention (3–14 days in duration), along with key results in terms of cardiorespiratory fitness, metabolic changes, body composition and any mechanistic measurements.

Reference	Participants	Inactivity	Effects			
			Cardiorespiratory fitness	Metabolic	Body composition	Mechanistic
Bowden Davies et al.^28^ 14 days SR; 14 days resuming activity	45 young adults (37 years, 27 BMI) <2 h ex/w 4 days screening	Activity by SenseWear. Steps/day ↓: BL 12,780, SR 2495 ↔ Habitual diet	Treadmill V̇O_2_ peak: ↓ 0.2 ml/min ↓ 2.2 ml/min/kg	OGTT: ↑ insulin AUC ↓ peripheral insulin sensitivity ↔ hepatic insulin resistance	DXA and MRS: ↑ fat mass, central and liver ↓ leg lean mass ↔ arms or trunk	Not yet reported
Olsen et al.^30^ 14 days SR	Young men <2 h ex/w 1 week screening Study 1: *n* = 8 (27 years, 23 BMI) Study 2: *n* = 0 (24 years, 22 BMI)	Activity by pedometer. Steps/day ↓: Study 1: BL 6203, SR 1394 Study 2: BL 10,501, SR 1344 ↔ Habitual diet	Not measured	OGTT: ↑ insulin AUC OFTT: ↑ insulin AUC, c-peptide and TG	DXA and MRI: ↔ fat mass ↑ intraabdominal fat ↓ fat-free mass	Not measured
Krogh-Madsen et al.^29^ 14 days SR	10 young men (24 years, 22 BMI) <2 h ex/w 1 week screening	Activity by pedometer and Actiheart. Steps/day ↓: BL 10,501, SR 1344 ↔ Habitual diet	Cycle V̇O_2_ max: ↓ 7.2% ml/min ↓ 6.6% ml/min/kg	H-E clamp: ↓ peripheral insulin sensitivity ↔ hepatic glucose production ↔ fasting bloods	DXA: ↔ fat mass ↓ leg lean mass ↔ arms or trunk	↓p-Akt/totalAkt at 4 h insulin stimulated ↔ mRNA
Knudsen et al.^26^ 14 days SR **+** overfeeding; 14 days resuming activity	9 young men (24 years, 22 BMI) 4 days screening	Activity by pedometer and Actiheart. Steps/day ↓: BL 10,278, SR 1521 Dietary intake ↑: 2762–4197 kcal/day	Cycle V̇O_2_ max: ↓3.8% ml/min ↓3.4% ml/min/kg	H-E clamp: ↓ peripheral insulin sensitivity ↔ hepatic glucose production 3 h OGTT: ↑ insulin AUC ↔ glucose AUC ↓ Matsuda ↔ fasting bloods ↑ leptin and adiponectin	DXA: ↑ total body mass, BMI, fat mass, android and gynoid ↔ fat-free mass MRI: ↑ visceral fat	Not measured
Dixon et al.^31^ 7 days SR	Middle aged men; 9 lean (52 years, 24 BMI) and 9 overweight (49 years, 29 BMI) 1 week screening	Activity by Actiheart. BL highly active >30 min 5/w, SR <4000 steps for 1 week. ↔ Habitual diet	Treadmill V̇O_2_ max (not reported pre and post)	OGTT: ↑ glucose and insulin AUCs ↑ TG but not HDL, LDL ↔ fasting bloods	DXA (not reported pre and post)	Not measured
Walhin et al.^27^ 7 days SR **+** overfeeding	26 young males (25 years, 24 BMI)	Activity by Actiheart. Steps/day ↓ with ↑ dietary intake +50% (SUR); additional subgroup with exercise (SUR+EX). SUR: 12,562 to 3520 and SUR+EX 10,544 to 3690 step/day	Treadmill V̇O_2_ max (not reported pre and post)	OGTT: ↑ SUR insulin AUC ↔ SUR+EX insulin AUC ↑ HOMA-IR	DXA: ↑ total body mass and lean mass ↔ fat mass	Altered expression of key genes and proteins in adipose tissue
Mikus et al.^32^ 3 days step reduction	12 young adults (29 years, 24 BMI) 1 week screening. Exclusion: ‘involved in competitive sporting events’	Activity by pedometer and EE/PA monitor. Steps/day ↓: BL 12,956, SR 4319 ↔ Habitual diet	V̇O_2_ max (not reported pre and post)	CGM: ↑ postprandial glucose ↔ pre-glucose or average OGTT: ↑ insulin AUC ↔ glucose AUC ↓ Matsuda ↑ HOMA-IR	DXA (not reported pre and post)	Not measured

AUC, area under curve; BL, baseline; BMI, body mass index; CGM, continuous glucose monitoring; DXA, dual-energy X-ray absorptiometry; EE, energy expenditure; EX, exercise; ex/w, exercise per week; h, hour; HDL, high-density lipoprotein; H-E, hyperinsulinemic–euglycemic; HOMA-IR, homeostatic model assessment of insulin resistance; LDL, low-density lipoprotein; MRI, magnetic resonance imaging; mRNA, messenger RNA; MRS, magnetic resonance spectroscopy; OGTT, oral glucose tolerance test; PA, physical activity; p-Akt, phosphorylated protein kinase B; SR, step reduction; SUR, energy surplus; TG, triglyceride; total Akt, total protein kinase B; V̇O_2_, maximum oxygen uptake; ↑ significant increase; ↓ significant decrease; ↔ did not significantly change.

Reduced ambulatory activity for 14 days demonstrates a significant accretion of adipose tissue and ectopic fat deposition. In the first study to communicate this finding (10 young, healthy males), intraabdominal fat mass significantly increased from 693 ml to 740 ml, with no changes in total body fat mass.^30^ Subsequent studies have reported findings in agreement; one example states an increase in body fat (predominately central) and magnetic resonance spectroscopy (MRS) measured liver fat was observed in 45 young, healthy males and females following a similar step-reduction protocol.^28^ Interestingly, individuals who were genetically predisposed to T2D (first-degree relatives) gained a greater relative amount of android (central compartment measured by dual-energy X-ray absorptiometry, or DXA) fat, when compared with those who were not. Given that central adiposity and liver fat deposition have been causally related to insulin resistance,^33^ it is perhaps unsurprising that glucose metabolism is also perturbed in all these studies, typically with maintenance of normal glucose profiles but hyperinsulinaemia.^28–30^ Insulin resistance has been primarily linked to impaired peripheral insulin resistance (inability of skeletal muscle to increase glucose uptake) rather than hepatic insulin resistance (an inability to inhibit endogenous glucose production) in both free-living^28,29^ and bed-rest^34^ models of reduced PA. Taken together, the evidence suggests that step reduction causes peripheral insulin resistance in skeletal muscle and adipose tissue, but while fat is accumulated centrally and in the liver, hepatic insulin resistance remains unchanged (in the short term).

**Figure 1.**
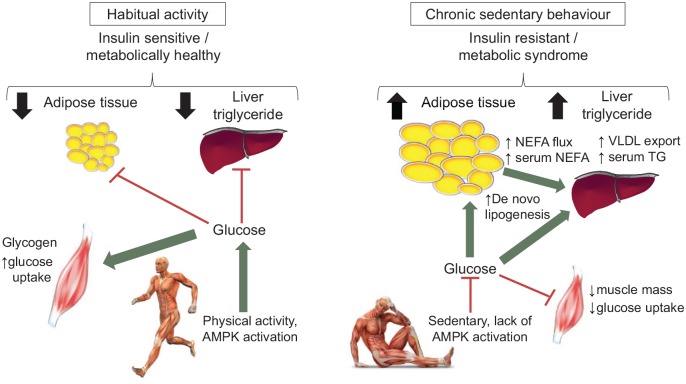
A two-part schematic representing the metabolic effects of habitual physical activity (left) and chronic sedentary behaviour (inactivity; right). Left: a consequence of sedentary behaviour is diminished AMPK activation and glucose uptake into skeletal muscle, inducing insulin resistance. The plasma glucose (not transported into muscle) provides a substrate for de novo lipogenesis in adipose tissue and liver. Consequently, there is expansion of adipose tissue mass, intrahepatic lipid accumulation and increased lipid export from the liver as VLDL triacylglycerol particles and serum triacylglycerol with induction of systemic insulin resistance. Right: being habitually active stimulates AMPK activation and glucose uptake into skeletal muscle; insulin sensitivity is therefore preserved and less glucose is diverted to metabolically unfavourable depots. AMPK, AMP-activated protein kinase; NEFA, nonesterified fatty acids; TG, triglyceride; VLDL, very low-density lipoproteins.

At a molecular level, 14 days of step reduction results in a significant decrease in insulin-simulated skeletal muscle Akt phosphorylation.^29^ Under normal physiological conditions, p-Akt would elicit a ‘downstream’ phosphorylation of AS160 (Akt substrate protein) after contractile activity to promote the translocation of GLUT4 glucose transporters to the plasma membrane. However, despite blunted p-Akt, AS160 did not significantly change but GLUT4 was not measured. Interestingly, plasma inflammatory markers such as TNF and IL-6 also did not change.^29^ A plausible explanation is that the short-term step-reduction protocol in healthy adults did not elicit a great enough stimulus for muscle inflammation and also offers some explanation for the dissociation between Akt/AS160. Walhin and colleagues found that 7 days of step reduction significantly altered the expression patterns of key metabolic genes involved with nutritional homeostasis, metabolism and insulin action (upregulation of SREBP-1C, FAS, GLUT4; downregulation of PDK4, IRS2, HSL) and the ratio between pAMPK/AMPK in adipose tissue.^27^ Interestingly, 45 min of daily exercise during energy-matched step reduction attenuated these changes. Collectively, this could suggest the following paradigm: a transition to physical inactivity causes a reduction in skeletal muscle insulin sensitivity, perhaps due to altered insulin signalling, contributing to a repartitioning of energy substrates into alternative tissues, increasing central fat accumulation and ectopic storage within the liver and other organs, exacerbating insulin resistance (Figure 1).^35,36^ As peripheral insulin resistance progresses, continued ectopic fat accumulation within the liver and pancreas precipitates development of the metabolic syndrome, a progressive decline in beta cell function and, ultimately, T2D.^37^

## Ageing and musculoskeletal health

Maintenance of musculoskeletal health is multifactorial, affected by lifestyle (e.g. nutritional status), biological (e.g. inflammation) and psychosocial factors (e.g. fear of falling).^38^ As well as the obvious stimulatory effects of feeding, physical activity/exercise is essential in preserving muscle mass; both amino acids and contractile activity represent major stimuli for muscle protein synthesis, which is the primary regulated variable influencing muscle mass. Previous guidelines recommended 0.8 g/kg of dietary protein intake per day for all individuals; revised consensus guidelines from sarcopenia working groups now recommend 1–1.2 g/kg per day in individuals over the age of 65.^39^ A combination of exercise training and dietary supplementation with essential amino acids (EAA) has been shown to be synergistic for increasing muscle protein synthesis (MPS).^40^

**Figure 2.**
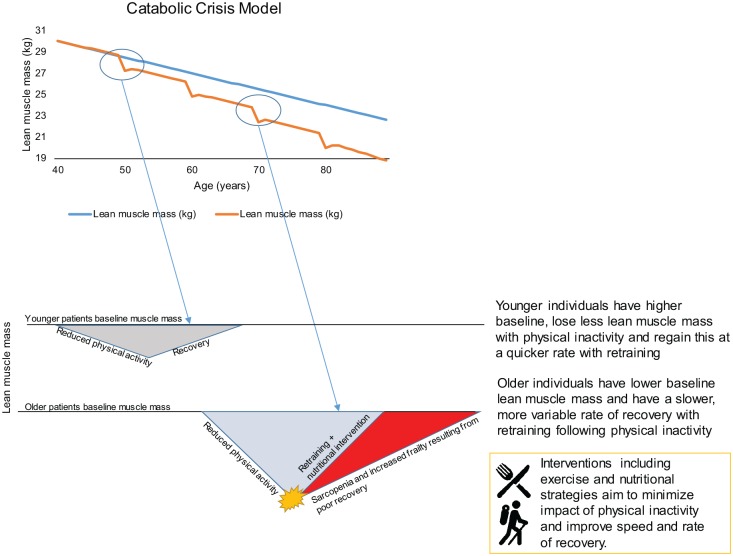
A two-part schematic representing the Catabolic Crisis model proposed by English and Paddon-Jones^47^ (upper figure) and the reduced activity models (young *versus* old) proposed by Perkin and colleagues^48^ (lower figure). Upper: the Catabolic Crisis model proposes that rather than the traditional linear model of age-related muscle loss (sarcopenia), instead episodes of acute illness or injury can accelerate muscle loss (indicated as a nadir on the graph) and are followed by periods of incomplete recovery. Lower: the reduced activity model suggests that older individuals compared to younger individuals tend to have less muscle mass and may lose muscle mass at a quicker rate (when subject to periods of inactivity), and recovery may be more variable. These two theories contextualize the importance of avoiding periods of prolonged inactivity, particularly in older adults.

Skeletal muscle mass declines with age at ~10% per decade over the age of 50 years, although with significant heterogeneity relating to both genetic and lifestyle factors.^41^ Sarcopenia, defined as a loss of muscle strength/power with ageing due to changes in the quality and quantity of muscle tissue, becomes increasingly prevalent with advancing age; 13–24% under 70 years but >50% over 80 years.^42^ While both muscle mass and muscle function are important for the health and well-being of older adults, the loss of muscle mass traditionally precedes decrements in muscle function and thus could represent an early sign of a potential progressive slide into frailty.^41^ Therefore, it is important to understand the cause of, and mitigating factors towards, this loss of muscle tissue with age. While it has traditionally been viewed of as an insipid, linear loss of muscle mass and/or function, the *Catabolic Crisis model* proposes that these ‘linear’ age-related changes may actually be punctuated and/or accelerated by intermittent periods of immobility associated with ill health and/or lifestyle changes (Figure 2). Importantly, a resumption of normal PA after a period of reduced activity may not restore normal glucose metabolism and, in the case of muscle loss, rates of MPS in older adults,^43^ which highlights the deleterious and persistent negative effects of inactivity in this population. Nevertheless, a decline in muscle mass and/or quality may precipitate a loss of an older adult’s physiological reserve, which could subsequently increase the risk of falls and fractures, reduce functional independence and predispose them to more hospital admissions and ultimately spells of sedentary behaviour.^44^ Hence, sarcopenia is associated with physical disability and impaired quality of life while the concomitant poor functional ability is associated with reduced survival.^44–46^

Cuthbertson and colleagues described the phenomenon of *anabolic resistance*, whereby skeletal muscle of older adults has a decreased sensitivity and responsiveness of MPS to EAA compared to that of younger adults, as a primary contributing factor for the age-related loss of muscle mass.^49^ At a molecular level, this is associated with decrements in the expression and activity of components of anabolic signalling pathways. While the anabolic resistance of ageing is likely multifactorial, it is well established that extreme models of inactivity (i.e. bed rest and limb immobilization) impair muscle protein metabolism and contribute to significant and rapid decreases in muscle mass.^50^ For example, Paddon-Jones and colleagues showed that young, healthy adults lose 2% of their muscle mass with 28 days of bed rest.^51^ However, this effect is more pronounced in the older population, with Kortebein and colleagues showing a 7% decrease in just 10 days of bed rest.^52^ Aside from older patients having lower baseline muscle mass, bed rest in older people is associated with an accelerated rate of loss of muscle mass with more variable recovery rates.^41^ To put this in context, the decline in muscle mass after 10 days bed rest is equivalent to 7 years of age-related sarcopenia.^41,52^ These extreme models of physical inactivity clearly demonstrated that a lack of muscle contractile activity is a precipitating cause of ‘anabolic resistance’ and muscle atrophy. However, the utility of these models for explaining the impact of low PA, that is ~2000–9000 steps per day^53,54^ in the average older adult, may need to be revisited.

## Musculoskeletal effects of inactivity/step reduction

Studies exploring the effects of step reduction on muscle protein turnover have focused on MPS rather than muscle breakdown as decreased MPS is generally regarded as the dominant mechanism causing more prolonged (i.e. >10 days) disuse muscle atrophy.^55^ The few studies conducted in older adults are summarized in Table 2. Although physical inactivity is associated with ~7% reduction in V̇O_2_ peak in healthy young adults, no data are available from the studies of older adults.^28,29^ Significant muscle atrophy is seen with only 14 days of step reduction in both young and older adults;^28,29,56^ the reported 1–4% losses in muscle mass are both striking and concerning, considering that sarcopenic muscle loss is estimated to occur at ~0.8% per year.^50^

**Table 2.** A summary of intervention studies examining the effects of reduced physical activity and increased sedentary behaviour in younger adults (>65 years old). The table outlines the participant cohorts (including age, BMI), details of the inactivity intervention (3–14 days in duration), along with key results in terms of muscle function, metabolic changes, body composition and any muscle protein turnover.

Reference	Participants	Inactivity	Effects			
			Muscle function	Metabolic	Body composition	Muscle protein turnover
Breen et al.^56^ 14 days SR	10 older adults (72 years, 29 BMI) >3500 step/day 3 days screening	Activity by SenseWear. Steps/day ↓: BL 5962, SR 1413 ↔ Habitual diet	↔ Muscle strength (isometric MVC) ↔ Physical function (SPPB)	OGTT: ↑ glucose and insulin AUCs ↓ Matsuda ↑ HOMA-IR ↑ TNF-α and CRP ↔ IL-6 and C-peptide	DXA: ↔ total body mass and total fat mass ↑ trunk fat mass ↓ leg fat-free mass	↓ 26% postprandial MPS ↔ basal and postabsorptive MPS ↔ intramuscular signalling proteins
Devries et al.^57^ 14 days SR + RT ± nutritional supplement	30 older men (70 years, 27 BMI) >3500 step/day 3 groups (*n* = 10)	Activity by SenseWear. Steps/day ↓: BL 6273–7714, SR 1161–1288	↔ Muscle strength (isometric MVC) SR ↔ Muscle strength (1RM) SR but ↑ in SR + RT	Metabolic assessments were made but not compared to BL	DXA: ↔ total fat mass/% and total fat-free mass ↓ leg fat-free mass SR	MPS measured but not compared against BL. Nutritional supplement had no effect. MPS lower in SR than SR + RT.
McGlory et al.^43^ 14 days SR; 14 days resuming activity	22 overweight prediabetic older adults (69 years, 27 BMI) >3500 step/day 1 week screening	Activity by SenseWear. Steps/day ↓: BL 7362 SR 991 ↔ Habitual diet	↔ Muscle strength (isometric MVC)	OGTT: ↑ glucose and insulin AUCs ↓ Matsuda ↑ HOMA-IR ↑ TNF-α, CRP and IL-6	DXA: ↔ total body fat % and lean mass	↓ 12% MPS, did not restore after resuming activity

1RM, one-repetition maximum; AUC, area under curve; BL, baseline; BMI, body mass index; CRP, C-reactive protein; DXA, dual-energy X-ray absorptiometry; h, hour; HOMA-IR, homeostatic model assessment of insulin resistance; IL-6, interleukin 6; MPS, muscle protein synthesis; MVC, maximum voluntary contraction; OGTT, oral glucose tolerance test; RT, resistance training; SPPB, short physical performance battery; SR, step reduction; TNF-α, tumour necrosis factor-alpha; w, week; ↑ significant increase; ↓ significant decrease; ↔ did not significantly change.

It was first demonstrated that an acute bout of walking (45 min or the equivalent of ~5000 steps) was sufficient to increase the anabolic sensitivity of older skeletal muscle to dietary amino acids,^58^ suggesting a significant factor in the ‘normal’ age-related anabolic resistance may be related to the level of habitual PA. In order to more clearly define the role of inactivity (but not complete immobilization) on postprandial rates of MPS, Breen and colleagues^56^ employed a step-reduction protocol whereby 10 healthy older adults (age 72 ± 1 year) restricted their daily steps from ~6000/day to ~1500/day for 14 days. Although there was no impact of this step reduction on basal rates of myofibrillar protein synthesis, the feeding-induced stimulation of myofibrillar protein synthesis after ingestion of 25 g of protein was blunted by ~26% after the 14 days. This inactivity-induced anabolic resistance was also accompanied by the progression of insulin resistance, evidence of systemic low-grade inflammation (i.e. increased C-reactive protein and tumour necrosis factor-α) and a loss of ~0.6 kg of leg fat-free mass in only 2 weeks,^56^ the latter of which was confirmed in a similar step-reduction study in older adults.^57^ Although there was no impact on muscle function (i.e. isometric strength or short physical performance battery score) in either study,^56,57^ the loss of muscle mass, which typically precedes reductions in muscle function,^59^ suggests this ‘benign’ form of inactivity could be an accelerating factor in the development and progression of sarcopenia.

**Figure 3.**
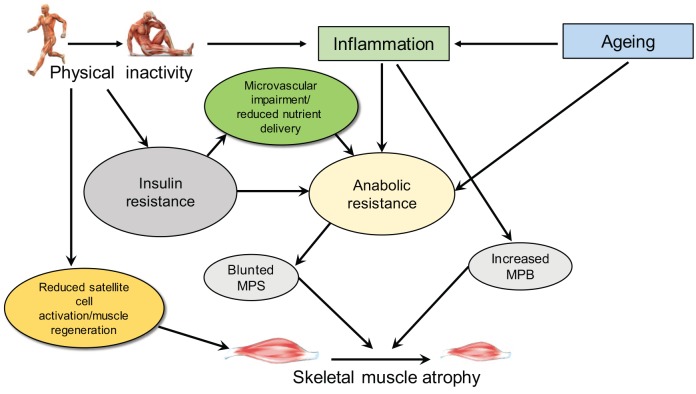
A schematic to summarize the reported effects of physical inactivity on skeletal muscle atrophy. Physical inactivity and ageing have both been linked with increased inflammation and anabolic resistance; microvascular impairment also has a role due to insulin resistance; and with blunted MPS and increased MPB skeletal muscle atrophy is exacerbated. Physical inactivity can also cause reduced satellite cell activation, also linked to atrophy. MPB, muscle protein breakdown; MPS, muscle protein synthesis.

The coincidence of muscle loss and body fat accumulation with ageing has led to the term ‘sarcopenic obesity’, which may present additional complications for the development of anabolic resistance and subsequent muscle loss.^60^ For instance, obesity alone has been associated with a blunted muscle protein synthetic response to dietary protein ingestion,^61^ although this finding is not universal.^62^ However, cross-sectional data suggest that inactivity in conjunction with obesity can exacerbate the anabolic resistance of ageing.^63^ This was further demonstrated in an elegant study by McGlory and colleagues,^64^ who examined the impact of 2 weeks of physical inactivity (i.e. reduction in daily steps from ~6500/day to ~1300/day) in overweight, prediabetic older men and women. The 14 days of step reduction were associated with a ~12% reduction in free-living rates of myofibrillar protein synthesis, which may reflect both a reduced stimulus for muscle remodelling (i.e. inactivity) and an attenuated free-living postprandial muscle protein synthetic response. While there was no detectable change in leg lean mass or muscle fibre cross-sectional area (CSA), the sustained suppression of integrated rates of MPS on resumption of habitual PA would be a concern for the subsequent development and/or progression of sarcopenia. The effects of PA/ inactivity on skeletal muscle protein turnover are summarized in Figure 3.

## Countermeasures during periods of step reduction

Acute metabolic^65^ and free-living^66^ studies provide a rationale for amino acid/protein supplementation to support MPS in older adults. However, the ability to enhance muscle mass and/or function with the chronic consumption of protein/amino acid-based supplements is somewhat equivocal in otherwise healthy adults,^67–69^ which is generally related to the heterogeneity of study designs and populations. Theoretically, exercise training eliciting sufficient anabolic adaptions, combined with efficient nutritional strategies, would be synergistic for maintaining muscle mass in older populations. However, not all populations will be capable of exercise interventions, and in these subpopulations efficient nutritional strategies alone may minimize loss in muscle mass.

A variety of countermeasures involving exercise/PA and nutritional strategies have been tested for their ability to protect individuals from deconditioning and attenuate inactivity-induced muscle atrophy. Studies in habitual settings and bed rest studies have been reviewed.^69,70^ This section of the review focuses on the few studies that have investigated countermeasures during acute step reduction, which are summarized in Table 3.

**Table 3.** A summary of intervention studies examining the effects of reduced physical activity with countermeasures to reduce metabolic and musculoskeletal effects. The table outlines the participant cohorts (including age, BMI), details of the inactivity intervention and countermeasure used, along with key results.

Reference	Participants	Inactivity	Countermeasure	Main findings
Moore et al.^71^ 14 days SR + RT	14 male older adults (71 year, 25% body fat) >3500 step/day	Activity by SenseWear. Steps/day ↓: BL 7011, SR <1500	SR only *versus* SR+EX: 6 sessions of unilateral low-load, high-effort resistance exercise (i.e. three sessions/week with at least 48 h between sessions) with a randomly selected leg	RT associated with greater muscle fibre CSA, satellite content and capillarization.
Devries et al.^57^* 14 days SR + RT ± nutritional supplement	30 older men (70 years, 27 BMI) >3500 step/day 3 groups (1) whey isolate, (2) micellar-whey, (3) micellar-whey + citrulline (*n* = 10 each group)	Activity by SenseWear. Steps/day ↓: BL 6273–7714, SR 1161–1288	Dietary intervention during SR. Throughout SR, participants performed 6 sessions of unilateral low-load, high-effort resistance exercise (3 sessions/week with at least 48 h between sessions) with a randomly selected leg	MPS similar across groups so dietary groups were collapsed to compare SR and SR+RT legs. MPS lower in SR than SR+RT in postabsorptive and postprandial states.
Walhin et al.^27^* 7 days SR **+** overfeeding ± aerobic exercise	26 young males (25 year, 24 BMI)	Activity by Actiheart. BL highly active >30 min 3× week, Steps/day ↓ with ↑ dietary intake +50% (SUR); additional subgroup with exercise (SUR+EX). SUR: 12,562–3520 and SUR+EX 10,544–3690 step/day	SUR (*n* = 14) *versus* SUR+EX (*n* = 12): 45 min of daily treadmill running at 70% of maximum oxygen uptake	Vigorous-intensity exercise counteracted most effects of short-term overfeeding and under-activity at whole-body level and in adipose tissue, despite standardized energy surplus
Perkin et al.^48^ 14 days SR + exercise	30 older men (aged 65–80 years)	Activity by pedometer. Steps/day ↓: BL >3500, SR <1500	SR only (*n* = 10) *versus* Progressive RT group (*n* = 10) *versus* ‘Exercise snacking’ home-based group (*n* = 10)	Not yet reported

BL, baseline; BMI, body mass index; CSA, cross-sectional area; EX, exercise; h, hour; MPS, muscle protein synthesis; RT, resistance training; SR, step reduction; SUR, energy surplus; * studies also listed in Tables 1 and 2 but described in a different context; ↑ significant increase; ↓ significant decrease.

### Effects of exercise during step reduction

Walhin and colleagues assessed the impact of 45 min of daily aerobic exercise, compared with no additional exercise, on participants undergoing a period of step reduction: the addition of the daily aerobic exercise prevented changes in insulin sensitivity, although energy expenditure and caloric intake remained matched between the two groups.^27^ In a single study conducted by Devries and colleagues^57^ and Moore and colleagues^71^ the effects of a unilateral resistance training protocol in older adults undergoing 14 days of step reduction was examined. The within-participant model compared low-load resistance exercise (three sessions per week) in a randomly selected exercised leg with the contralateral leg that did not exercise. Six sessions of low-load, high-effort resistance exercise during 14 days of step reduction maintained leg lean mass, muscle function and a robust feeding-induced rise in MPS in postabsorptive and postprandial states.^57^ Further, the exercised leg had greater muscle fibre CSA, satellite cell content and capillarization.^71^ It was suggested that these results are due to the ability of low-load, high-effort resistance exercise to enhance motor unit (and thus muscle fibre) recruitment.^72^ Taken together these studies highlight the importance of performing exercise even during brief periods of physical inactivity for preserving anabolic and insulin sensitivity, and thus muscle mass and metabolic function. Studies looking at different types of concomitant exercise during periods of step reduction in older men (resistance training prior to step reduction, exercise three times per day during the step-reduction period and a control) are also under way.^48^

### Effects of nutritional strategies during step reduction

The study by Devries and colleagues^57^ also examined whether citrulline, as an arginine and nitric oxide precursor, could attenuate muscle anabolic resistance accompanying step reduction. Participants were randomized to one of three dietary intervention groups (*n* = 10 per group) differentiated by the type of protein and free amino acids included in the product: (1) ‘whey isolate’ − 5 g glycine/day during step reduction and 20 g isolated whey protein plus 15 g glycine on the infusion trial day; (2) ‘micellar-whey’ − 5 g glycine/day during step reduction and 20 g micellar-whey protein plus 15 g glycine on the infusion trial day; and (3) ‘micellar-whey + citrulline’ − 5 g citrulline/day during step reduction and 20 g micellar-whey protein plus 5 g citrulline on the infusion trial day. None of the nutritional strategies induced a differential MPS following step reduction. Based on these results and previous work,^73^ citrulline does not act to increase MPS in healthy older adults at rest, following exercise or following a period of inactivity.

## Areas for further investigation, and public health implications

The studies reviewed here typically recruit small samples of healthy adults that have only minimally investigated age and gender-specific differences, which may limit the clinical generalizability of the results on the impact of inactivity in acute or chronic illness. In clinical populations we might expect to observe a greater decompensation, making these changes more clinically significant. In patients who already have metabolic complications (e.g. NAFLD, T2D), the impact of step reduction might lead to changes that would be less easily reversed and contribute to further metabolic decline. Equally, older adults who have suboptimal musculoskeletal health might see similar effects. Alarmingly, data from 239 older adults (~77 years) admitted to hospital with an acute illness reported that mean daily step count was only 740,^74^ supporting the experimental design reviewed here. The longest duration of a step-reduction study is 14 days; studies of greater duration would be more relevant to the wider population, given the chronic nature of sedentary behaviour, although the practical implementation of such a protocol in a research setting is challenging. In clinical trials including control groups that are physically inactive, progressive metabolic deterioration was apparent; however, meeting current PA guidelines was associated with preventing this.^75^ Furthermore, higher levels of PA have been positively associated with improved sleep quality^76^ and insomnia,^77^ which are linked to metabolic outcomes. There is still much to be understood about the maladaptations to sedentary behaviour, but this should be a key research priority for health care providers and policy makers.

## Summary

The largest public health gains are potentially from encouraging very sedentary people (in society or a clinical setting) to be more physically active, along with education about the health risks of sedentary behaviours (such as prolonged screen time). Effective and practical measures must be developed to counteract the deleterious metabolic and musculoskeletal effects of recurrent or chronic periods of physical inactivity.
